# Super impact stable TATB explosives recrystallized by bicarbonate ionic liquids with a record solubility

**DOI:** 10.1038/s41598-020-61470-9

**Published:** 2020-03-11

**Authors:** Wen-Li Yuan, Guo-Hong Tao, Lei Zhang, Zhang Zhang, Ying Xue, Ling He, Jinglun Huang, Weifei Yu

**Affiliations:** 1 College of Chemistry, Sichuan University, Chengdu, 610064 China; 2 Institute of Chemical Materials, China Academy of Engineering Physics, Mianyang, 621999 China

**Keywords:** Ionic liquids, Organic molecules in materials science, Characterization and analytical techniques, Design, synthesis and processing

## Abstract

Ensuring the security for long-term storage of weapons is always of the great cMehilaloncerns in the field of energetic materials. 1,3,5-Triamino-2,4,6-trinitrobenzene (TATB) is a remarkable explosive applied in nuclear weapons where extreme safety is required primarily. Owing to the strong inter or intra molecular hydrogen bonding, TATB shows poor solubility in most solvents. As the result, the particle shape and size of TATB products is hard to regulate, which closely related to the weapons stability. Herein, a new recrystallization method is provided to refine TATB using bicarbonate ionic liquids. Bicarbonate ionic liquids exhibited the record solubility (26.7 wt%) for dissolving TATB explosive. The recrystallized TATB were spherical particles with uniform size and showed extremely insensitivity to impact (>100 J) and friction (>360 N). Moreover, the experimental ^1^H and ^13^C NMR spectra of TATB in solution are reported for the first time.

## Introduction

It is well known that traditional energetic compounds like 2,4,6-trinitrotoluene (TNT) and 1,3,5-trinitro-1,3,5-triazine (RDX) are cheap, easily prepared with good performance. However, most classical energetic materials are not qualified for strategic weapons because of their unsatisfactory mechanical stability. This apparent defect is an important reason leading to the Palomares H-bomb accident and Thule air base nuclear weapon accident, when 6 of the 8 nuclear bombs detonated by air crash, causing more than 2000 m^3^ water and 20 hectares of land polluted by plutonium^1–4^. These pollutions are considered as the most serious nuclear weapon accidents ever occurred^5–7^. For this reason, the related militaries and governments invested a great deal of effort to ensure the safety of strategic weapons. In 1970s, a new type of energetic materials called insensitive high explosives (IHE) were developed and applicated^8,9^. Among them, 1,3,5-triamino-2,4,6-trinitrobenzene (TATB) is the most attractive and famous IHE. TATB has many unique advantages including high density, low toxicity and low vapor evaporation, which are suitable for long-term storage of the strategic weapons^10,11^.

Recrystallization is a favorable approach to increasing the quality of energetic compounds, for instance, improving the stability of 1,3,5,7-tetranitro-1,3,5,7-tetrazocane (HMX)^12^. This method is also of great significance for improving the stability of TATB and ensuring the safety of strategic weapons. Similar to many insoluble hydrogen-bond-rich materials^13,14^, TATB has strong intramolecular and intermolecular hydrogen bonding interactions, leading to a closely packed structure. Thus, TATB is insoluble in common solvents, causing a series refining problems during its production and application^15,16^. Dimethyl sulfoxide (DMSO) is currently used for recrystallizing TATB. Whereas, solubility of TATB in DMSO is too low (0.7 wt%) in room temperature and the solution need be heated at least 135 °C before carrying out^17^. Concentrated sulphuric acid (H_2_SO_4_) can dissolve TATB up to 20 wt%^18^, no significant improvement on quality of TATB can be assured after refinement^19^. For other nucleophilic polar solvents like 40% NaOH solutions or ammonia (NH_3_ H_2_O), new impurities such as 1,3,5-trihydroxy-2,4,6-trinitrobenzene (THTNB) lower the sensitivity of recrystallized TATB products^20^. Besides, these harsh recrystallization solvents are also a challenge to equipment, and harmful to environment and human body. So, mild solvents with high solubility for refining TATB is much looking forward to.

Compared with molecular solvents, ionic liquids are green designable materials exhibiting unique physical and chemical behaviors^21–23^. The task-specific ionic liquids have many advantages in dissolution and extraction^24,25^. Ionic liquids have been reported to dissolve a series of hydrogen-bond-rich natural products and 2D layered materials including cellulose, chitin, WS_2_, and graphene, showing their great potential in biomass, catalysis, solar cell and semiconductor^21–27^. For this reason, finding a kind of ionic liquid as TATB recrystallizing solvent will overcome the shortcomings of molecular solvents. Therefore, developing new kinds of ionic liquids to solve TATB’s refining problem is of great interesting in the field of energetic materials.

**Figure 1:**
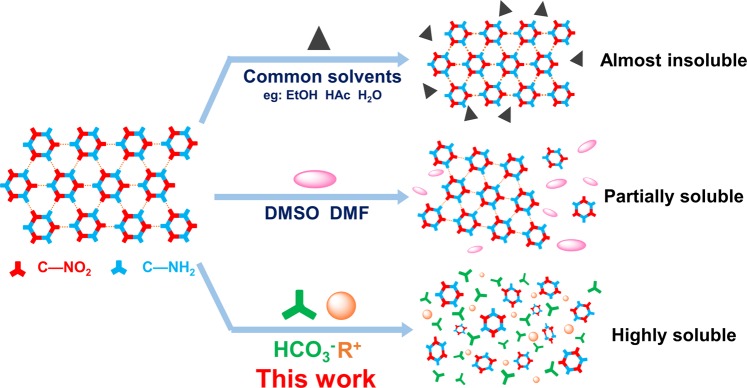
Dissolving ability and behaviour of TATB explosive in known solvents.

In this work, we first investigated new materials for dissolving and refining TATB explosive based on 1-butyl-3-methylimidazolium bicarbonate (BmimHCO_3_) and N,N,N,N-tetrapropylammonium bicarbonate (N_3333_HCO_3_) ionic liquids. (Fig. 1) BmimHCO_3_ and N_3333_HCO_3_ ionic liquids are weak alkaline and colorless liquids at room temperature. By virtue of high TATB solubility in bicarbonate ionic liquids, the dissolution behaviour and structure of TATB in solutions were systematically studied. The morphology of raw TATB and recrystallized TATB in shape and size are discussed. In addition, sensitivity test of recrystallized TATB products were conducted and showed much more insensitive against impact and friction. This recrystallization method is helpful to improve the safety of TATB and gives more strategies for dissolving hydrogen-bond-rich materials.

## Results and Discussion

### Solubility of TATB in BmimHCO_3_ and N_3333_HCO_3_ ionic liquids

**Figure 2:**
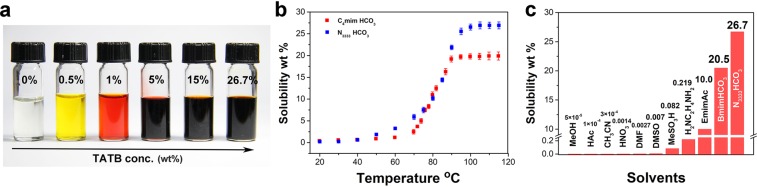
(**a**) Solutions of TATB in N_3333_HCO_3_ ionic liquid. (from 0 wt% to saturated (26.7 wt%)). (**b**) Dissolution curves of TATB in N_3333_HCO_3_ from 20 °C to 115 °C. (**c**) Solubility of TATB in common solvents and bicarbonate ionic liquids.

The solubility is measured by UV-Vis spectrophotometer from room temperature to 115 °C. (Fig. 2b) BmimHCO_3_ and N_3333_HCO_3_ can dissolve 0.4 wt% and 0.18 wt% TATB at 20 °C, respectively. The color of solutions varied from colorless to yellow. When the solutions were heated up to 70 °C, TATB’s solubility prominently increased, reaching 2.5 wt% and 5.9 wt% in BmimHCO_3_ and N_3333_HCO_3_, separately. And the solutions turned into dark red. The solubility of TATB reaches the maximum when the temperature exceeds 100 °C. These bicarbonate ionic liquids dissolve much more TATB than any solvent reported. BmimHCO_3_ can dissolve 20.7 wt% TATB at 110 °C, which is far higher than the known maximum record, 10 wt% in EmimAc^17^. Especially, N_3333_HCO_3_ exhibits the highest solubility for TATB, reaching 26.7 wt% at 105 °C, and setting a new soluble record for TATB. The solubility of TATB in bicarbonate ionic liquids are clearly higher than that of common solvents, DMSO (0.007 wt%) and DMF (0.0027 wt%) currently in use. Bicarbonate ionic liquids also have a higher solubility than the harsh solvents including concentrated H_2_SO_4_ (20 wt%) and NaOH (6.1 wt%)

### Spectroscopic investigation of TATB in bicarbonate ionic liquids

**Figure 3:**
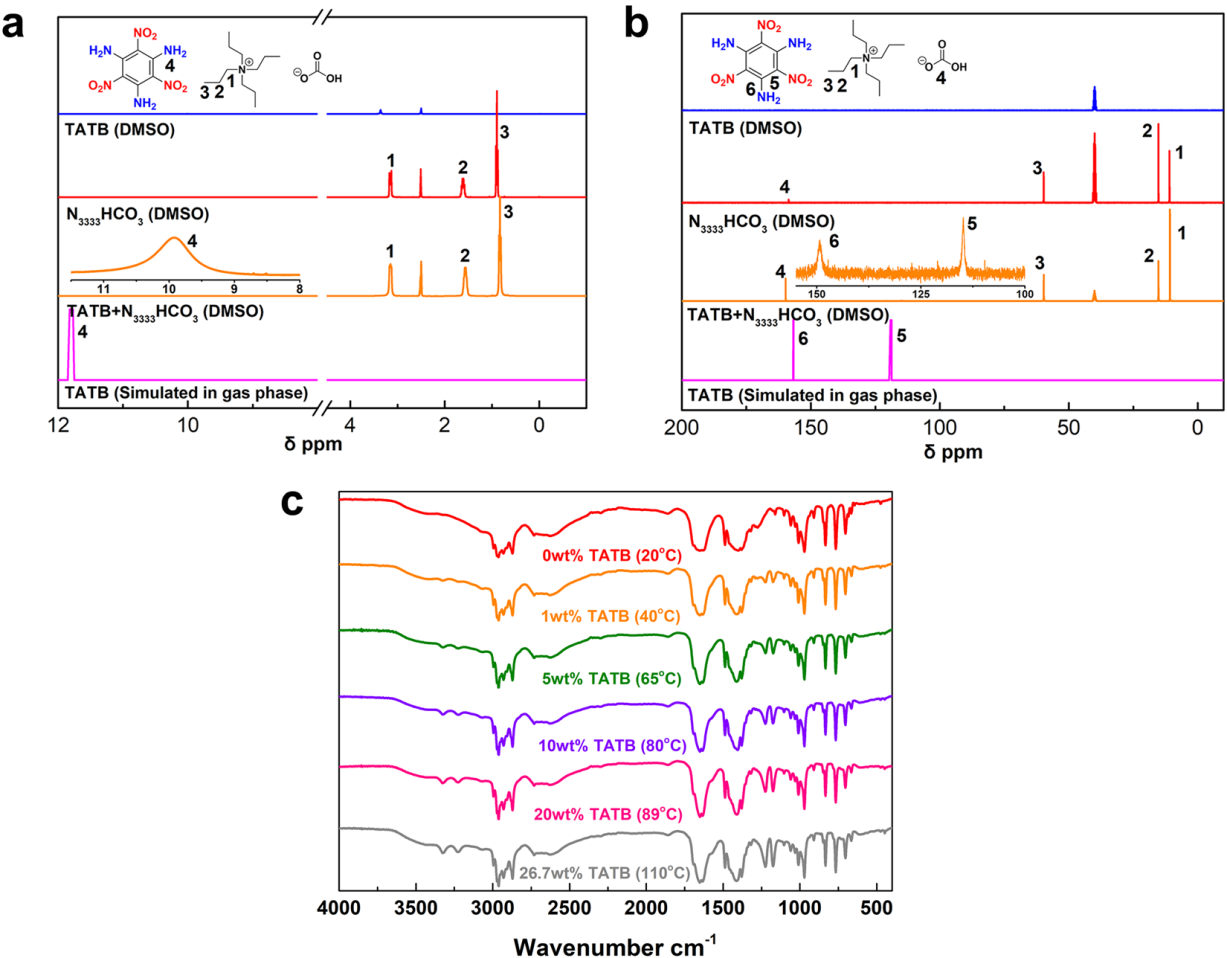
^1^H NMR spectra (**a**) and ^13^C NMR spectra (**b**) of TATB in neat DMSO-*d*_6_ and N_3333_HCO_3_ solution with DMSO-*d*_6_ as locking solvent. (**c**) FTIR spectra of saturated TATB solutions at different temperatures.

Due to the insoluble nature of TATB, no experimental NMR spectra of TATB was reported since its initial synthesis in 1887, which makes the structural research on TATB highly limited. In this work, the ^1^H and ^13^C NMR spectra of TATB in solution were collected for the first time. (Fig. 3a,b) No any signal according to TATB in DMSO-*d*_6_ is found without bicarbonate ionic liquids. However, obvious typical peaks of TATB are recorded on both ^1^H and ^13^C NMR spectra in N_3333_HCO_3_ with DMSO-*d*_6_ as locking solvent. The peak locates at 169.20 ppm on ^13^C spectrum is attributed to the central sp^2^ carbon atom in bicarbonate anion. With the addition of N_3333_HCO_3_, a new signal on ^1^H spectrum located at 9.88 ppm and two signals at 148.65 ppm and 114.22 ppm on ^13^C spectrum are clearly observed. These signals belong to the aromatic ring of TATB and coincide with the theoretical prediction of TATB in gas phase.

To better understand the solubility behaviour of TATB in bicarbonate ionic liquids, IR spectra of saturated TATB in N_3333_HCO_3_ solutions were obtained at different temperatures. (Fig. 3c) The specific frequencies of recrystallized TATB coincide with the standard TATB spectrum^28^, where 1219 cm^−1^ and 1175 cm^−1^ are attributed to the C-N stretch of amino and nitro groups connected to the aromatic ring, and the symmetric and asymmetric vibrations of N-H bonds are located at 3218 and 3317 cm^−1^. Moreover, the absorption intensity of C-N bond and N-H bond is enhanced, suggesting the concentration of TATB increases. The characteristic absorptions of recrystallized TATB in C-N and N-H bond do not shift in solutions at different temperatures, which can be confirmed by previous literature and Spectral Database for Organic Compounds (SDBS)^28,29^. Besides, the IR spectra of N_3333_HCO_3_ as solvent did not have obvious change from room temperature to 110 °C. TATB maintains its own structure and spectral properties in bicarbonate ionic liquids, which are different from other nitrobenzene intermediates^30^.

**Figure 4:**
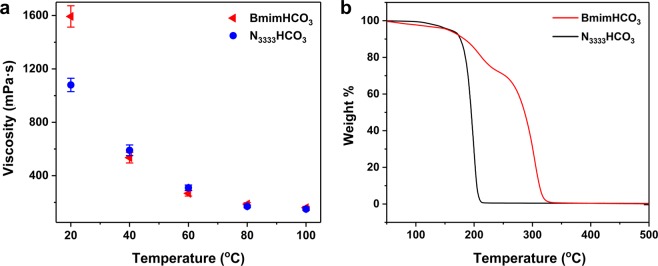
(**a**) Viscosities of BmimHCO_3_ and N_3333_HCO_3_ ionic liquids from 20 °C to 100 °C. (**b**) TGA curves of BmimHCO_3_ and N_3333_HCO_3_ ionic liquids.

We also investigated the viscosity of bicarbonate ionic liquids under the standard atmospheric pressure (Fig. 4a). Owing to the formation of bicarbonate anion dimer is thermodynamically favored, pure bicarbonate ionic liquids exhibit a large viscosity higher than 1000 mPa·s in room temperature. When heated up to 100 °C, the viscosity of BmimHCO_3_ and N_3333_HCO_3_ are decreased obviously to 187 mPa·s and 171 mPa·s, respectively. Compared with the common recrystallization solvents like DMSO (1.99 mPa·s) and H_2_SO_4_ (23.8 mPa·s), BmimHCO_3_ and N_3333_HCO_3_ are still in a higher viscosity but have no effect on TATB dissolution in bicarbonate ionic liquids, which means the solvation of TATB is dominated by other physicochemical parameters of the solvents.

Thermal stability of bicarbonate ionic liquids is of vital importance in TATB recrystallization, which directly affect the yield and purity of refined TATB explosives. The thermal stabilities of BmimHCO_3_ and N_3333_HCO_3_ ionic liquids were assessed by thermogravimetric analysis (TGA) (Fig. 4b). TGA results show that their initial weight reduction temperatures are higher than 170 °C, and no significant weight loss before 120 °C. The bicarbonate ionic liquids are also determined by differential scanning calorimetry (DSC). No obvious chemical decomposition is observed before 130 °C. The endothermic peak is found in 185 °C and 190 °C of BmimHCO_3_, N_3333_HCO_3_, coinciding with the above TGA results. All the ionic liquids maintain their chemical properties under 130 °C and provide stable solvent environment for refining TATB explosive.

**Figure 5:**
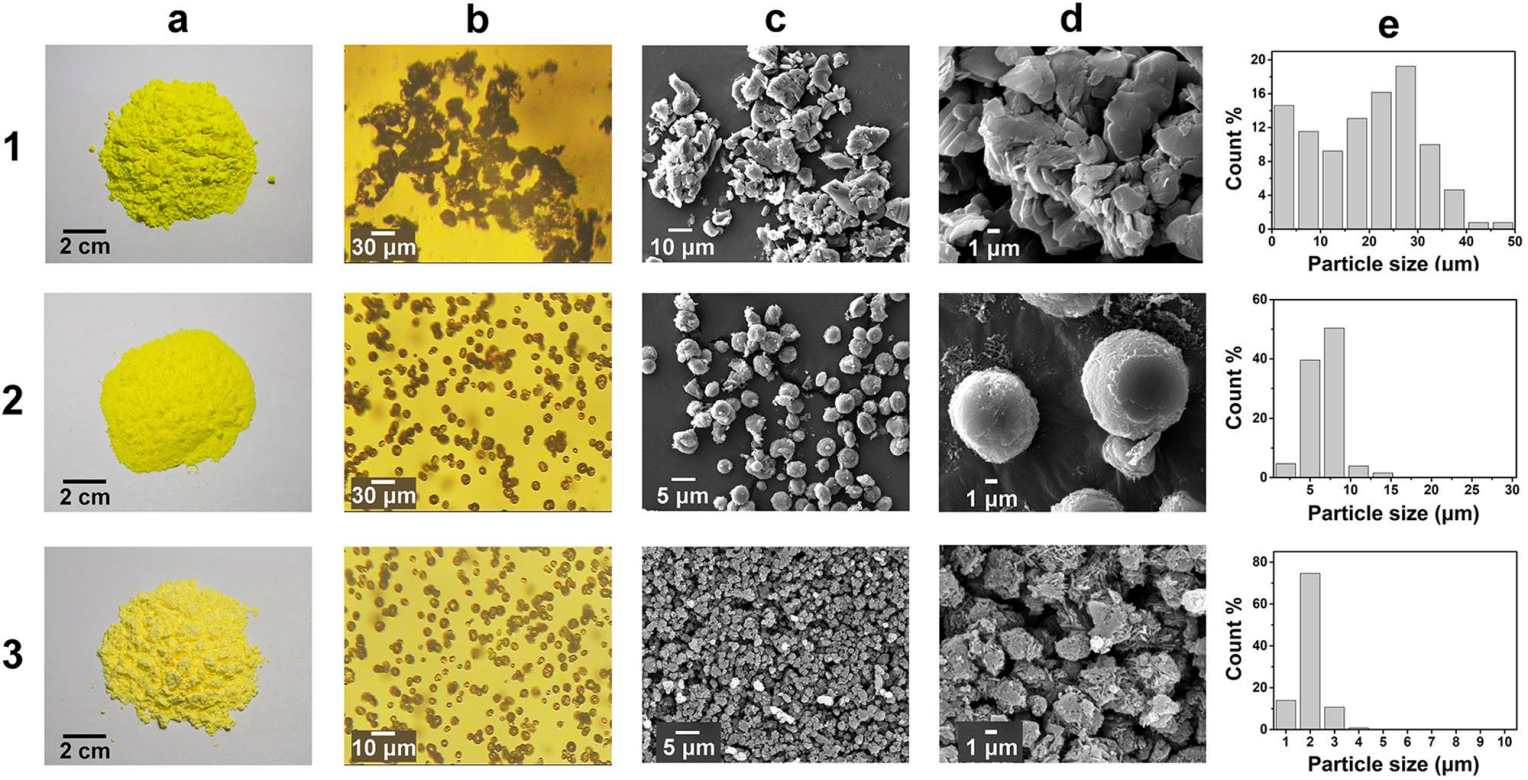
Morphological characterization of TATB with (1) untreated, (2) recrystallized by BmimHCO_3_ and (3) recrystallized by N_3333_HCO_3_. (**a**) Macroscopic observation of TATB explosive. (**b**) Microscopy image of TATB particles. (**c**) SEM images of TATB particles. (**d**) High resolution SEM image of TATB particles. (**e**) Particle size distribution histograms of TATB explosive.

The particle shape and size of energetic compounds are closely related to the sensitivity. We analyzed the morphology and particle size of raw and recrystallized TATB by optical microscopy (OM) and scanning electron microscopy (SEM). (Fig. 5) The appearance color of two TATB products (2a, 3a) after recrystallization do not show any significant change compared to raw TATB (1a). Different form the irregular accumulated particles of raw TATB under the OM (1b), homogeneously dispersed spherical particles of recrystallized TATB (2b) and (3b) are clearly observed. The N_3333_HCO_3_ recrystallized TATB exhibits smaller particles size under the higher magnification. Angular and defective particles composed of tightly packed flaky crystals are observed in the raw TATB (1c, 1d). In comparison, refined TATB (2c, 2d) exhibited smooth sphere and uniform size after recrystallized by BmimHCO_3_, and the TATB product (3c, 3d) has smaller particles with regular size distribution after recrystallization by N_3333_HCO_3_. Different orientation of crystalline growth is an important reason causing obvious defects of raw TATB. Furthermore, the size distribution histograms were performed in column (e) in Fig. 5. The raw TATB (1e) has a wider distribution from 1.0 μm to 50 μm, among which 78% of TATB are below 30 μm. The recrystallized TATB 2 shows a narrower particle size distribution from 5.0 μm to 10 μm, and the N_3333_HCO_3_ recrystallized TATB 3 displays much more regular particle size within 2.0 ± 0.5 μm. Bicarbonate ionic liquids make TATB a smaller size and a more regular morphology.

Common TATB explosive owns a low mechanical sensitivity with 50 J to impact and >360 N to friction. (Table 1) Lowering impact sensitivity of TATB is still of vital importance for IHE. A noticeable result is that the recrystallized TATB in bicarbonate ionic liquids shows an unusual mechanical insensitivity (impact sensitivity >100 J and friction sensitivity >360 N). Compared to the mechanical sensitivity of raw TATB explosive and other TATB products recrystallized by EmimAc/DMSO^31^, the TATB recrystallized by BmimHCO_3_ and N_3333_HCO_3_ are much more insensitive against impact. This phenomenon is attributed to the improved morphology of recrystallized TATB, which can be explained by hot spot theory of Bowden *et al*.^32^. Hot spots can be reduced by modifying the particles shape, surface roughness and size of energetic compounds. The defective particles in raw TATB are modified by recrystallization of bicarbonate ionic liquids, which can reduce the quantity of hot spots generated by external stimulus. Besides, with uniform particle surface and particle size, recrystallized TATB particles can lower the probability of hot spots and disperse the localized heat in time.

**Table 1:** Summary of solubility and sensitivity of TATB.

Products/Methods	Solubility (wt%)	Size (μm)	H50 (cm)^[a]^	IS (J)^[b]^	FS (N)^[c]^
Raw TATB	—	4–35	162	50	>360
ATK^[d]^	—	28–77	>177	—	>360
BAE^[d]^	—	5	>177	—	>360
Directly synthesized by NH_3_ H_2_O^[d]^	—	5–60	>177	—	>360
Directly synthesized by NH_3_^[d]^	—	20–35	>177	—	>360
EmimAc/DMSO^[e]^	10	0.052–0.066	125	50	>360
DMSO^[f]^	2.5	300–2000	—	—	—
Conc. H_2_SO_4_^[g]^	20	2–5	—	—	—
Conc. NaOH^[h]^	6.1	1.71–5.03	—	—	—
BmimHCO_3_	20.5	5–8	>320	>100	>360
N_3333_HCO_3_	26.7	1.5–2.5	>320	>100	>360

^[a]^50% impact height. ^[b]^Impact sensitivity. ^[c]^Friction sensitivity. ^[d]^Ref. ^39^. ^[e]^Ref. ^29,40^. ^[f]^Ref. ^41^ ^[g]^Ref. ^31^. ^[h]^Ref. ^42^.

## Conclusion

In conclusion, we provided a new strategy to greatly improve the solubility and impact stability of TATB explosive. The super impact intensive TATB was obtained by recrystallization using BmimHCO_3_ and N_3333_HCO_3_ ionic liquids. The superior TATB has great potential for replacing existing TATB products, and it will make the weapons more secure. Furthermore, this method provides a new idea for dissolving the insoluble hydrogen-bond-rich materials. After obtaining high concentration solutions of hydrogen-bond-rich materials, NMR analysis could be introduced to study more details on their structures and properties.

## Experimental Section

### General methods

All chemical reagents were purchased in analytical grade. IR were recorded on Bruker ALPHA infrared spectrometer by using attenuated total reflection (ATR) mode. ^1^H and ^13^C NMR spectra are recorded by Bruker AVANCE III HD with magnetic intensity 9.4 T and resonance frequency of 400 MHz and 100 MHz respectively. The samples are dissolved in locking solvent *d*_6_-DMSO and their chemical shifts are analyzed and recorded in ppm by compared with the internal standard tetramethylsilane (TMS). Differential scanning calorimetry (DSC) test were taken on TA Q20 calorimeter with nitrogen as shield gas and indium as standard. The temperatures were recorded from −80 °C to 400 °C in a heading speed of 10° min^−1^. Thermogravimetric analysis (TGA) were finished on NETZSCH TG 209F1 with nitrogen as shield gas of 70 mL min^−1^ and heating from 25 to 500 °C at 0.1 MPa in 10 °C min^−1^ Hydrogen, carbon and nitrogen elemental analyses is finished by Elementar Vario MICRO CUBE instrument. Schimadzu-xrd-6100 recorded PXRD data from 5° to 80 ° in a rate of 0.2°/min. Microscopic morphologies of the TATB were taken by HLP-85C polarizing microscope. The SEM test were obtained by Hitachi TM3000. Sensitivity were test by BCJ drop hammer testing machine and BMC friction testing machine.

### Theoretical study

The computations were taken by the Gaussian09 (Revision A.02) programs to analysis the geometric optimization and frequencies of molecules by using functional group B3LYP and basis set 6-311 + G(2d,p)^33,34^. Single-point energies of all the molecules were also calculated at the level of B3LYP/6-311 + G(2d,p)^35,36^. The optimized structures were get at the local energy minima on their potential energy surfaces and without any imaginary frequency. The NMR analysis results of TATB molecule was shown and analyzed by GaussView 5.0 program^37^.

### Sensitivity test

According to the UN Recommendations on the Transport of Dangerous Goods-Tests and Criteria^38^, the sensitivity was performed by BAM method. The sample of raw TATB and recrystallized TATB is enclosed in a special impact device which receiving and transferring the energy by drop weight. The impact sensitivity of explosive can be defined as the lowest impact energy leading to the explosive decomposed from at least one out of at least six trials. Impact energy is calculated from the drop weight (kg), the acceleration of gravity (m/s^2^) and the fall height (m). If the sample at lower energy is no reaction, the test is continued with increased impact energies until the explosion is observed firstly. As for friction sensitivity test, the sample is test between the porcelain plate and peg at a load up to 360 N. The friction sensitivity of explosive can be defined as the highest friction load leading to the explosive decomposed from at least one out of at least six trials. If the sample at highest friction load (360 N) is no reaction, the result should be recorded as >360 N.

### 1-Butyl-3-methylimidazolium bicarbonate (BmimHCO_3_)

Solid NaOH (400 mg, 10 mmol) was added in batches into 20 mL ethanol solution of 1-butyl-3-methylimidazolium bromide (2.19 g, 10 mmol) in Schlenk tube. The resulting mixture was stirred in N_2_ atmosphere at room temperature for 4 h. A colorless solution was gotten after filtered the precipitate. Excess carbon dioxide was bubbled into the solution in air at room temperature. After removing the ethanol solvent and dried in the vacuum for 24 hours, a pale-yellow liquid of BmimHCO_3_ was obtained in 83% yield. ^1^H NMR (400 MHz, *d*_6_-DMSO): *δ* = 9.30 (s, 1H), 7.85 (s, 1H), 7.77 (s, 1H), 4.20 (d, 2H), 3.88 (s, 3H), 1.78 (m, 2H), 1.26 (m, 2H), 0.90 (t, 3H) ppm. ^13^C NMR (100 MHz, d6-DMSO): *δ* = 167.45, 136.49, 123.53, 122.22, 48.40, 39.52, 35.74, 31.33, 18.72, 13.24. IR (ATR): *ν* = 3420 (m), 3082 (s), 2959 (vs), 2868 (s), 2733 (w), 1701 (m), 1620 (s), 1566 (vs), 1459 (s), 1422 (m), 1373 (m), 1333 (m), 1307 (w), 1279 (w), 1248 (w), 1212 (w), 1159 (vs), 1134 (m), 1117 (m), 1083 (m), 1051 (m), 1013 (m), 956 (w), 891 (s), 835 (w), 808 (s), 753 (s), 699 (m), 655 (s), 631 (s), 412 (m).

### N,N,N,N-tetrapropylammonium bicarbonate (N_3333_HCO_3_)

Solid NaOH (400 mg, 10 mmol) was added in batches into 20 mL ethanol solution of N,N,N,N-tetrapropylammonium chloride (2.22 g, 10 mmol) in Schlenk tube. The resulting mixture was stirred in N_2_ atmosphere at room temperature for 4 h. A colorless solution was gotten after filtered the precipitate. Excess carbon dioxide was bubbled into the solution in air at room temperature. After removing the solution and dried in the vacuum for 24 hours, a colorless liquid of N_3333_HCO_3_ was obtained in 87% yield. ^1^H NMR (400 MHz, *d*_6_-DMSO): *δ* = 3.15 (s, 2H), 1.63 (s, 2H), 0.90 (s, 3H). ^13^C NMR (100 MHz, d6-DMSO): *δ* = 158.10, 59.26, 14.80, 10.53. IR (ATR): *ν* = 3397 (m), 2962 (s), 2931 (s), 2879 (s), 2735 (m), 2674 (m), 1855 (m), 1641 (vs), 1490 (s), 1460 (s), 1381 (vs), 1368 (vs), 1334 (s), 1274 (m), 1189 (w), 1159 (w), 1105 (m), 1060 (s), 1031 (s), 1008 (s), 972 (vs), 908 (w), 835 (s), 768 (s), 683 (s), 645 (w).

## Supplementary information

Supplementary Information.

## Data Availability

The authors declare that the data supporting the findings of this study are available within the Supplementary Information files.
