# Supplementary material for: Super impact stable TATB explosives recrystallized by bicarbonate ionic liquids with a record solubility
Source: Sci Rep. 2020 Mar 11;10:4477. doi: 10.1038/s41598-020-61470-9 (PMC7066152; doi:10.1038/s41598-020-61470-9)
Supplement: Supplementary file 1 — Supplementary Information. [file 41598_2020_61470_MOESM1_ESM.docx]

Supplementary Information

Super impact stable TATB explosives recrystallized by bicarbonate ionic liquids with a record solubility

Wen-Li Yuan, Guo-Hong Tao, Lei Zhang, Zhang Zhang, Ying Xue, Ling He*, Jinglun Huang and Weifei Yu*

College of Chemistry, Sichuan University, Chengdu 610064, China.

Institute of Chemical Materials, China Academy of Engineering Physics, Mianyang, 621999, China.

E-mail address: lhe@scu.edu.cn, yuwf_1988@caep.cn

Index:

S2 NMR spectra of bicarbonate ionic liquids

S4 FTIR spectra of bicarbonate ionic liquids, raw TATB and recrystallized TATB explosives

S5 PXRD patterns and DSC curves of raw TATB and recrystallized TATB explosives

S6 Predicted NMR spectra of TATB

S7 Sensitivity tests of raw TATB and recrystallized TATB explosives

**Figure S1** NMR spectra of bicarbonate ionic liquids.

^1^H NMR spectrum of 1-butyl-3-methylimidazolium bicarbonate ionic liquids.


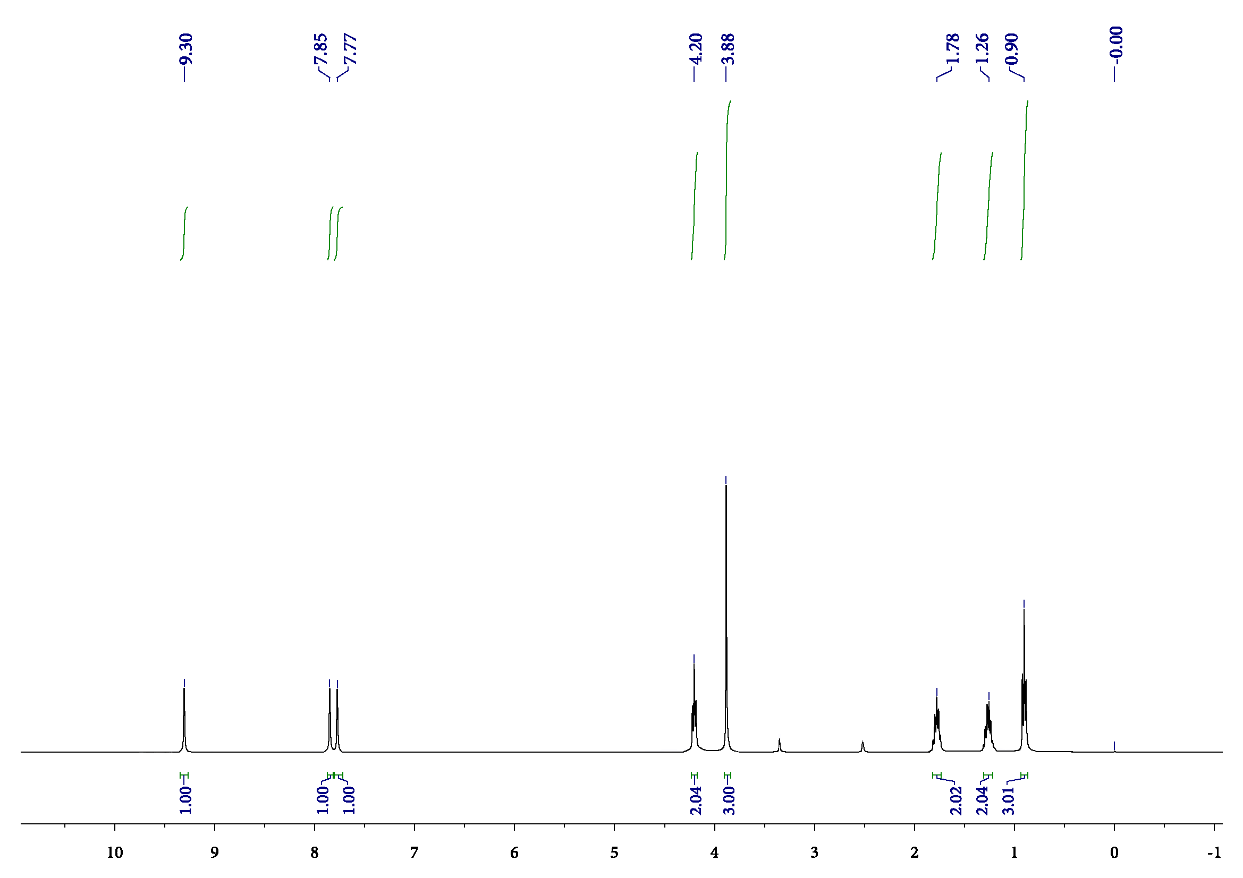


^1^H NMR spectrum of N,N,N,N-tetrapropylammonium bicarbonate ionic liquids.


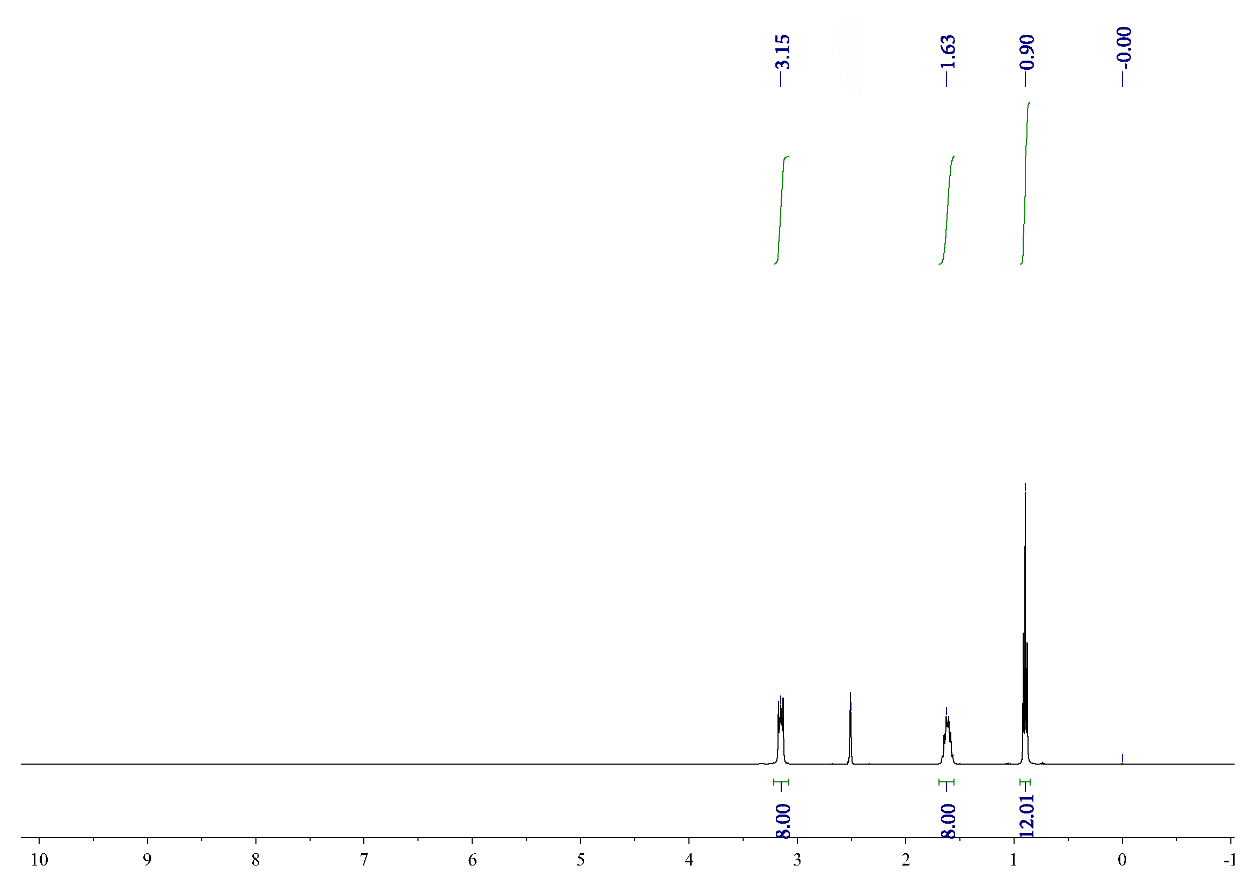


^13^C NMR spectrum of N,N,N,N-tetrapropylammonium bicarbonate ionic liquids.

**
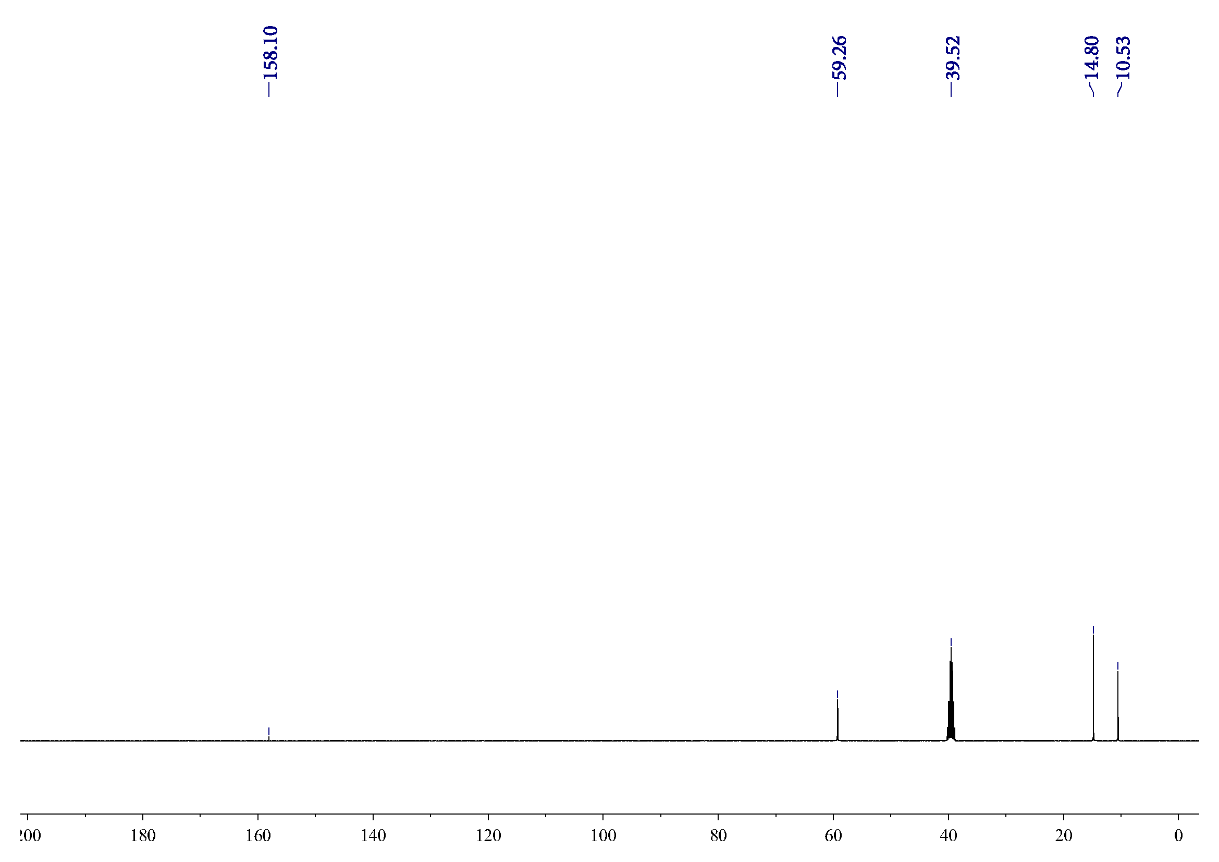
**

^13^C NMR spectrum of 1,2-dimethyl-3-ethylimidazolium bicarbonate ionic liquids.


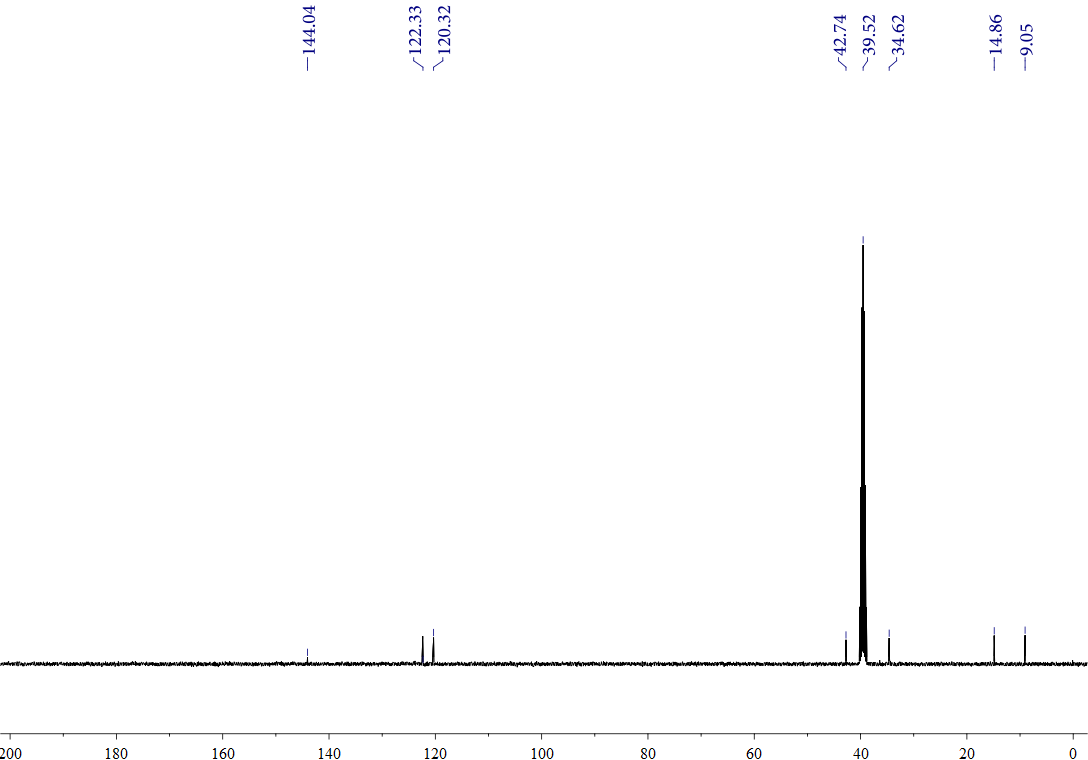


**Figure S2** FTIR spectra of bicarbonate ionic liquids





**Figure S3** FTIR spectra of raw TATB and recrystallized TATB explosives





**Figure S4** PXRD patterns of raw TATB and recrystallized TATB explosives





**Figure S5** DSC curves of raw TATB and recrystallized TATB explosives





**Figure S6** Predicted ^1^H NMR spectrum of TATB in gas phase.





**Figure S7** Predicted ^13^C NMR spectrum of TATB in gas phase.





**Table S1** Sensitivity tests of raw TATB and recrystallized TATB explosives

Friction test of TATB under 360 N

|  | 1 | 2 | 3 | 4 | 5 | 6 | 7 | 8 | 9 | 10 |
| --- | --- | --- | --- | --- | --- | --- | --- | --- | --- | --- |
| raw TATB | - | - | - | - | - | - | - | - | - | - |
| recrystallized TATB (BmimHCO_3_) | - | - | - | - | - | - | - | - | - | - |
| recrystallized TATB (N_3333_HCO_3_) | - | - | - | - | - | - | - | - | - | - |

Impact test of TATB under 50 J

|  | 1 | 2 | 3 | 4 | 5 | 6 | 7 | 8 | 9 | 10 |
| --- | --- | --- | --- | --- | --- | --- | --- | --- | --- | --- |
| raw TATB | + | + | + | - | - | + | - | + | - | + |
| recrystallized TATB (BmimHCO_3_) | - | - | - | - | - | - | - | - | - | - |
| recrystallized TATB (N_3333_HCO_3_) | - | - | - | - | - | - | - | - | - | - |

Impact test of TATB under 100 J

|  | 1 | 2 | 3 | 4 | 5 | 6 | 7 | 8 | 9 | 10 |
| --- | --- | --- | --- | --- | --- | --- | --- | --- | --- | --- |
| raw TATB | + | + | + | + | + | + | + | + | + | + |
| recrystallized TATB (BmimHCO_3_) | - | - | - | - | - | - | - | - | - | - |
| recrystallized TATB (N_3333_HCO_3_) | - | - | - | - | - | - | - | - | - | - |
